# A protocol for the process evaluation of a multi-centre randomised trial to compare the effectiveness of geriatrician-led admission avoidance hospital at home versus inpatient admission

**DOI:** 10.1186/s13063-018-2929-4

**Published:** 2018-10-19

**Authors:** Petra Mäkelä, Mary Godfrey, Andrea Cradduck-Bamford, Graham Ellis, Sasha Shepperd

**Affiliations:** 1 Nuffield Department of Population Health, Richard Doll Building, University of Oxford, Old Road Campus, Oxford, OX3 7LF UK; 2 Institute of Health Sciences, University of Leeds, Leeds, LS2 9LJ UK; 3 Monklands Hospital, Monkscourt Avenue, Airdrie, ML6 0JS UK; 4 London School of Hygiene and Tropical Medicine, Keppel Street, London, UK

**Keywords:** Process evaluation, Comprehensive Geriatric Assessment, Hospital at home, Admission avoidance

## Abstract

**Background:**

Attempts to design services to support the delivery of healthcare closer to home have taken various forms as countries respond to an increase in hospital admission rates for older people, who are at risk of hospital-acquired morbidity, prolonged lengths of stay and readmission. Evidence to support the development of these services is limited. We are conducting a process evaluation, alongside a UK multi-site randomised trial, to understand the contexts and practices of implementing geriatrician-led admission avoidance hospital at home services and to explore ways that the intervention might be effective, under what conditions, for whom, and how it differs from inpatient care.

**Methods:**

We are interviewing patients and their caregivers, from sites that are purposively sampled from participating National Health Service (NHS) trusts across the UK. We are also visiting sites to observe local processes and discuss the establishment and running of services with a range of multidisciplinary staff, managers, commissioners, primary care and social services representatives. We aim to interview approximately 36 patients and their caregivers with experience of hospital at home or inpatient services; 12 at each of three sites. We will use a content analysis approach to explore data across participants, services and sites.

**Discussion:**

This process evaluation will enable evaluation of implementation processes prior to knowing trial outcomes. We encompass domains of reach, delivery, change, context and response to the intervention by patients, their carers, health professionals and the health system.

**Trial registration:**

ISRCTN60477865. Registered on 10 March 2014. Trial sponsor: University of Oxford.

Version 3.1, registered on 14 June 2016.

## Background

The demographic change of population ageing has increased the demand for healthcare services internationally [1, 2]. Older people who present to healthcare services with an acute event may be admitted to hospital in the absence of alternative responses [3]. Older people often have complex needs and, when experiencing a health event, are vulnerable to adverse outcomes, a state commonly referred to as frailty [4–6]. As an inpatient, an older person with frailty may contend with hospital-acquired morbidity, a prolonged length of stay and a likelihood of readmission to hospital [4, 7–9].

In the UK, an emphasis on avoiding admission to hospital has gained momentum alongside an annual rise in emergency admissions [10, 11]. Growing demands for costly inpatient care and an increasingly constrained budget for the National Health Service (NHS) [12] have led to a policy focus on moving care away from hospitals to the community [3, 11, 13, 14]. Geriatrician-led admission avoidance hospital at home (HAH) is a model that is emerging internationally, referring to hospital-level treatment that is delivered by healthcare professionals (sometimes as a hospital outreach service) to patients who remain in their own homes [15, 16]. This is guided by the principles of Comprehensive Geriatric Assessment (CGA), a cornerstone of care for older people requiring hospital-level healthcare [17–19]. CGA is defined as ‘a multidimensional, multidisciplinary process which identifies medical, social and functional needs, and the development of an integrated/coordinated care plan to meet those needs’ [20].

We describe the methods of a process evaluation that we are conducting alongside a multi-site randomised trial of geriatrician-led admission avoidance HAH compared with hospital admission [21]. The main outcome will be ‘living at home’ at 6 months’ follow-up and other outcomes will include the incidence of delirium, mortality, new long-term residential care, cognitive impairment, activities of daily living, quality of life, quality-adjusted survival, length of stay, readmission or transfer to hospital and resource use. The randomised controlled trial (RCT) protocol has been described in detail previously [21].

## Aims and objectives

Within the context of a multi-site randomised trial we aim to expand understandings of what works in geriatrician-led HAH and inpatient settings, for whom, in what respects, to what extent, in what contexts, and how. The objectives are:

- To explore components, practices and experiences of CGA in HAH and inpatient settings
- To describe key elements of trial contexts and explore how these might affect implementation of the intervention and of the RCT
- To identify unanticipated consequences and aspects of the trial that are not necessarily captured quantitatively

## Methods

This a priori process evaluation is informed by the UK’s Medical Research Council (MRC) guidance [22, 23] and a framework for evaluation within trials [24]. Ethical approval for the trial and process evaluation was given by the Research Ethics Committee England, Wales and Northern Ireland (14/WA/1081) and Scotland (14/SS/1046).

### Setting

**Fig. 1:**
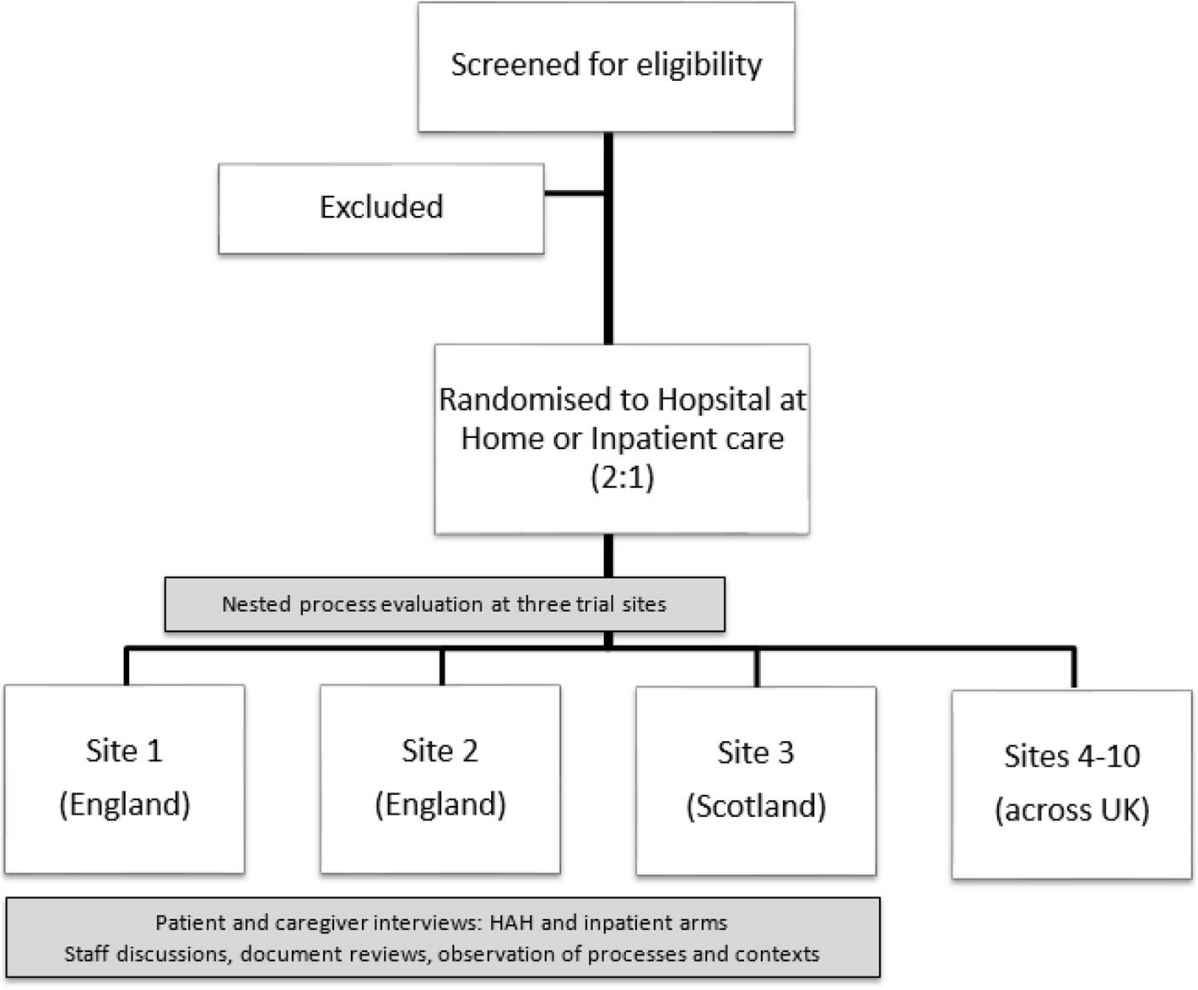
Process evaluation overview

We have selected three sites from those participating in a multi-site trial (two NHS trusts in England and one in Scotland, see Fig. 1) enabling comparison of implementation and a range of perspectives. These sites represent differing geographical areas, service compositions, populations and organisational arrangements that might influence the delivery and effectiveness of the intervention [23].

**Table 1:** Research questions

Domain	Focus
Context	• *Outer setting*: what is the local, regional and national context? How may social, political and economic contexts influence implementation? • *Inner setting*: how do organisational and service structures, cultures and relationships influence implementation?
Implementation	• What are the *characteristics of those delivering and receiving the intervention* in HAH and inpatient settings? • What are the *intervention characteristics* and *processes of implementation* in each setting, and how do these relate to the experiences and engagement of patients and caregivers? • How do these aspects of implementation relate to the existing programme theory and logic model for CGA (Fig. 2)?
Recruitment	• How are individuals recruited and by whom? • Are those recruited to the RCT representative of the overall ‘real world’ target population? • How do RCT implementation processes differ across settings? • How are RCT processes sustained or threatened over time? • Are there unintended consequences in processes and outcomes related to involvement in RCT, to the intervention or to other aspects of care?

*CGA* Comprehensive Geriatric Assessment, *HAH* hospital at home, *RCT* randomised controlled trial

### Design

The process evaluation design follows three interlinked stages: (1) determining existing theory and objectives underlying the complex intervention, (2) refining the research questions and (3) generating data.

#### Existing theory and objectives underlying the complex intervention

**Fig. 2:**
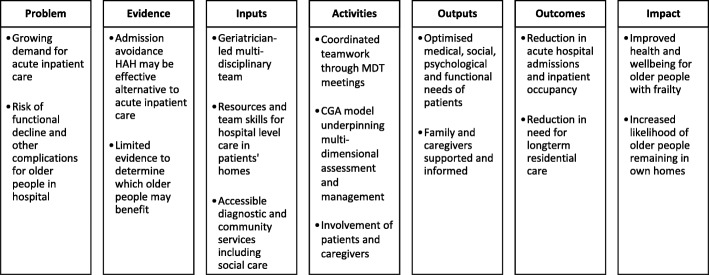
Simplified logic model for Comprehensive Geriatric Assessment (CGA)

A logic model of CGA (Fig. 2) outlines key aspects that may be shared across different settings and displays the relationship between resources, activities and intended results [25]. The accompanying programme theory proposes that implementation of CGA through geriatrician-led admission avoidance HAH, instead of an acute hospital admission, will lead to a greater improvement in health outcomes due to the hospital environment potentially limiting the processes of recovery.

#### Process evaluation questions

Our research questions address implementation of CGA in HAH and inpatient settings, and implementation of the RCT across trial contexts. We are guided by the Consolidated Framework for Implementation Research (CFIR) [26, 27], which specifies five key domains: intervention characteristics, outer setting (factors external to the organisation), inner setting (characteristics of the organisation), characteristics of the individuals involved in the intervention, and the processes of implementation [26] (see Table 1). Our intention is to undertake the process evaluation with sufficient flexibility to allow us to identify and address additional questions that may arise during the course of the study, recognising that local and broader aspects of these domains are likely to become more salient as the work iteratively develops [28, 29].

#### Generating data

We generate qualitative data to explore the research questions through field visits and by undertaking discussions with a range of multidisciplinary staff, managers, commissioners, representatives of primary care and social services. In addition, we conduct semi-structured interviews with a purposive sample of patients who have been randomised to the trial and their caregivers. Data generation is conducted iteratively over time to develop relationships with each site.

##### Field visits

Field visits are being conducted to find out about how the services are organised, the reach of the intervention, and interfaces with related services delivering health and social care. We review policy and guidance documents referred to by staff, service assessment documents, discharge templates, protocols or other documentation considered important to the services. We undertake in-depth discussions with a range of staff in both settings at each of the three sites, and capture field notes with the use of an observation framework attending to environment, ways of working, roles, interactions, relationships, activities, documents, methods of communication and other aspects that facilitate teamwork, clinical processes and patient or caregiver interactions.

##### Healthcare professionals

We purposively sample healthcare professionals to achieve diversity of roles and experience, aiming to yield ‘information-rich’ discussions [30]. In addition, we use snowball sampling where managers or clinical leads identify clinicians and support staff who are known to have suitable experience of the service, for whom brief ‘release’ from workload activities has been agreed for their participation in a discussion with the qualitative researcher [31]. Discussions are undertaken individually or with small groups, according to staff time constraints and availabilities. We aim to continue staff discussions until data generated enable interpretations and credible explanations that address the research questions [32].

Multidisciplinary staff invited to discussion at each of the three sites include managers and clinical leads for services participating in the RCT, allied health professionals across a range of seniorities, staff nurses, ward sisters and matrons, healthcare assistants, rehabilitation support workers, consultant geriatricians, junior doctors, physician assistants, primary care representatives, pharmacists, social workers, administrative and domestic services staff, and healthcare commissioners in England and Health and Social Care Partnerships in Scotland. We also discuss RCT implementation with principle investigators and research coordinators at each site. We capture aspects of variation between staffing complements; for example, whether teams include pharmacists or social workers. In addition, we anticipate that differences in team complements will be dynamic through the study and we explore contextual factors driving changes, vacancies or restructuring within services. Collection of contextual data will enable exploration of differences including associated service provision, perceptions of the intervention and drivers of change perceived in the healthcare environment.

##### Patients and caregivers

We invite a sample of patients randomised to HAH or inpatient care from each of the three sites to participate in qualitative interviews. We include people with and without family caregivers or formal carers and, where available, we invite family caregivers to take part in the interview also. We approach patients with cognitive or communication impairment, including people who may lack or have variable capacity to consent to the interview study, if they have appropriate support available to take part. Capacity assessment follows the principles of Mental Capacity Act (2005) in England [33] and the Adults with Incapacity Act (2000) in Scotland [34].

In view of the practicalities of arranging interviews in patients’ homes across geographically dispersed sites, and identifying patients who have been randomised to each arm of the RCT (with a 2:1 randomisation ratio for intervention to control), convenience sampling is also necessary [31]. The planned sample size is six patients (with a family member, caregiver or significant other), for each arm of the RCT, at each of the three sites (i.e. approximately 36 interviews in total). We are flexible to the potential need for a small number of further interviews to pursue promising lines of enquiry not anticipated in advance. We review the recruitment strategy iteratively, to ensure adequate sampling and to address practical issues that may affect timely access to participants.

The qualitative researcher approaches patients regarding the interview study following initial contact made by the trial research nurse or coordinator at the site, who confirms that patients are medically stable, are not receiving end of life care and that there are no other identified reasons why approach may be inappropriate or intrusive for the patient and family. The research nurse or coordinator provides written information about the interview study and confirms participants’ agreement for further contact. The qualitative researcher then discusses the interview study with the patient or consultee, answers questions and arranges a convenient time for the interview. The researcher arranges interviews around the time of discharge, or soon after discharge, from the healthcare service participating in the RCT, and carries these out in patients’ homes or on inpatient wards. We emphasise that patients and caregivers are not under any obligation to participate in the interview study and that, if they agree to take part, they are free to withdraw at any time without having to provide an explanation and without any effect on their usual care. We explain that all personal information will be anonymised and that researchers are not connected to patients’ clinical care.

#### Interview processes

The researcher records patient and caregiver interviews using an encrypted digital audio-recording device, subject to permission by participants. Interviews last between 30 and 60 min. We use topic guides as prompts for the semi-structured interviews, with versions adapted for experiences of HAH or inpatient care. We developed the topic guides from earlier iterations informed by focus group discussions with older people and family caregivers who had experience of HAH or admission to hospital. Within interviews, the researcher avoids the use of ‘jargon’ terms that may be unfamiliar to participants (such as ‘CGA’ [35]), but instead uses phrases that may illustrate aspects of experience; for example, 'Can you describe ways the healthcare team supported you?'. Areas of exploration within interviews include:

- Participants’ accounts of their presenting event and means of accessing acute healthcare
- Perceptions of interactions with healthcare professionals and other staff throughout the trajectory of service input for their presenting episode, from assessment to discharge and any follow-up received
- Whether any documentation was provided to patients and caregivers and, if so, how they perceived and used this (e.g. service information leaflets, goal sheets, medication information, discharge letters)
- How patients understood the intervention or other measures to have contributed to recovery from their presenting event and their ability to continue to manage after discharge
- Caregivers’ perceptions of positive and negative aspects of the healthcare experience and how effectively they perceived the patient’s and their own needs to have been addressed
- How and where they received input from healthcare services, and how healthcare professionals communicated or discussed transitions with them

We use a flexible approach and consider individuals’ specific healthcare experiences as conceived by participants themselves; for example, experiences that have extended to transitions between different parts of the health and social care system. The interview is adapted if topics appear to be causing undue distress. Where appropriate, the interview ceases and the researcher offers a subsequent interview date; for example, if events external to the interview have contributed to distress and if the participant would like to complete the discussion at a later stage. The researcher will follow up instances of distress with the site research team and principle investigator to determine any actions required.

#### Ethical issues

Patients who have been recruited to the RCT may have reduced, fluctuating or absent capacity to consent to trial participation and requirements [21]. As capacity to consent refers to a time- and decision-specific ability [36], capacity is assessed for inclusion in the interview study as an additional consideration to consent for overall RCT participation. If assessment indicates that a patient lacks capacity to consent to participate, the researcher discusses the interview study with a personal consultee who is ‘engaged in caring for the person or is interested in their welfare’ [33] and is involved in making decisions that reflect the patient’s views and values [37]. The researcher also invites the personal consultee to the interview study, following their informed consent, to share their own experiences and perspectives and to support the patient.

Personal consultees may support people with cognitive or communication impairment through the generation of ‘scaffolds’ in conversation, which can be used by the person in making sense of the topic under discussion, assisting them to find words or recognise reference points [38], p.341. We pay attention to use of terminology within interviews, particularly ways participants choose to refer to themselves and their respective roles, as questions that emphasise caregiving within families may differ from individuals’ usual ways of thinking about their mutual relationships [39]. As the majority of interviews take place in patients’ homes (or professionals’ places of work), the researcher becomes a ‘guest’ in these spaces and allows participants to set their own pace in discussions [40].

#### Reflexivity

The qualitative researcher captures observations and impressions in field notes soon after interactions and field visits, including contextual information and reflections on potential influences of the researcher’s own perspectives. [40, 41].

### Data management and analysis

Audio data is fully transcribed by a professional agency and is checked by the researcher for accuracy. We allocate pseudonyms to all participants and anonymise other potential identifiers within transcripts. All personally identifiable data is stored in password-protected files in a secure environment. Field notes pertaining to site visits and observations from interviews in home settings are managed anonymously and analysed. We use a spreadsheet to log all ‘raw’ data generated, to detail progress and highlight potential gaps in the evaluation. Data are subsequently managed in NVivo 11 (QSR international) to aid sorting and organising of the large volume and different types of data, and to facilitate analysis.

#### Analysis of contextual data

We will produce a narrative, descriptive account of the organisation of services at each site studied, drawing on data collated in field notes from observations during visits, review of formal documents relating to organisation and delivery, and notes from discussions with a range of healthcare professionals. We focus on factors guided by CIFR domains: intervention characteristics, outer setting (external to the organisation), inner setting (within the organisation), individuals involved and the processes of implementation. Changes to staffing and service organisation are captured, which might have impact on the delivery of healthcare. The evaluation of trial implementation explores sustainability of activities and strategies used at the interface between service delivery (in varied clinical settings) and RCT protocol requirements.

#### Analysis of qualitative interviews with patients and caregivers

We plan to undertake content analysis of transcribed interviews to interpret data through identifying, organising, describing and reporting themes or patterns [42, 43]. The analysis involves an iterative and reflective process that develops over time, moving back and forward between data generation and analysis, allowing issues identified to inform subsequent data generation and deepen areas of exploration.

We use a framework approach to facilitate the content analysis, address the research questions, and enable comparison within and between participants’ control and intervention experiences [44, 45]. We will develop an initial coding framework deductively from literature on CGA and HAH, the existing logic model and programme theory. We will expand this inductively from analysis of field notes and interviews and will develop the framework through iterative analysis across data sources, with ongoing discussion among the research group, aiming to capture new and unexpected issues that arise [44]. The framework approach to organising content analysis provides a clear trail of evidence for the credibility of the study [46], allowing management of large volumes of data while retaining connection to original sources, to avoid 'decontextualisation and fragmentation' of participants’ accounts [26], p.810. Extracts of raw data will be embedded within the interpretive narrative, to illustrate complexities that move beyond a description of data [43, 46].

### Dissemination

We will disseminate findings in a final report, peer-reviewed publications in academic and practitioner journals, and through conference presentations. We will engage with healthcare professionals’ and patient and caregivers’ groups to facilitate translation of findings, to inform clinical practice and to assist in contextualisation of trial findings.

## Discussion

This protocol outlines the design, data generation and analysis plan for a process evaluation embedded in a multi-centre trial of geriatrician-led admission avoidance HAH compared with hospital admission. The use of qualitative research facilitates understanding of how the intervention works in practice and aims to identify aspects perceived important by patients, caregivers and practitioners. Limitations of the process evaluation include prospective decisions about the focus within sites, though the design incorporates flexibility to respond to and expand research questions as the process evaluation progresses.

## Abbreviations

CFIR: Consolidated Framework for Implementation Research
CGA: Comprehensive Geriatric Assessment
HAH: Hospital at home
MDT: Multidisciplinary team
MRC: Medical Research Council
NHS: National Health Service
RCT: Randomised controlled trial
